# The HELP-UnaG Fusion Protein as a Bilirubin Biosensor: From Theory to Mature Technological Development

**DOI:** 10.3390/molecules30030439

**Published:** 2025-01-21

**Authors:** Paola Sist, Ranieri Urbani, Federica Tramer, Antonella Bandiera, Sabina Passamonti

**Affiliations:** 1 Department of Life Sciences, University of Trieste, 34127 Trieste, Italy; psist@units.it (P.S.); ftramer@units.it (F.T.); abandiera@units.it (A.B.); 2 Department of Chemical and Pharmaceutical Sciences, University of Trieste, 34127 Trieste, Italy; rurbani@units.it

**Keywords:** human elastin-like polypeptides, UnaG, recombinant fusion proteins, HUG, bilirubin, translational research, technology readiness levels

## Abstract

HUG is the HELP-UnaG recombinant fusion protein featuring the typical functions of both HELP and UnaG. In HUG, the HELP domain is a thermoresponsive human elastin-like polypeptide. It forms a shield enwrapping the UnaG domain that emits bilirubin-dependent fluorescence. Here, we recapitulate the technological development of this bifunctional synthetic protein from the theoretical background of its distinct protein moieties to the detailed characterization of its macromolecular and functional properties. These pieces of knowledge are the foundations for HUG production and application in the fluorometric analysis of bilirubin and its congeners, biliverdin and bilirubin glucuronide. These bile pigments are metabolites that arise from the catabolism of heme, the prosthetic group of cytochromes, hemoglobin and several other intracellular enzymes engaged in electron transfer, oxygen transport and protection against oxygen free radicals. The HUG assay is a powerful, user-friendly and affordable analytical tool that alone supports research at each level of complexity or taxonomy of living entities, from enzymology, cell biology and pathophysiology to veterinary and clinical sciences.

## 1. Introduction

HUG (acronym of HELP-UnaG) is a new recombinant fusion protein composed of two moieties, a human elastin-like polypeptide (HELP) carrier linked by a peptide bond to the N-terminal of UnaG [1]. HELP is an artificial polypeptide derived from the most regularly repeated motif of human elastin, resulting in an expression product with characteristic thermoresponsive properties [2]. UnaG is a natural protein first discovered in the muscle of the marine organism Japanese eel that binds the tetrapyrrolic compound bilirubin with high affinity emitting fluorescence [3]. HUG retains the functional properties of both parent proteins, though without identical parameters.

HUG was developed to address the need of a high-throughput, easy, affordable, sustainable and reliable analytical method to uniformly determine bilirubin and its congeners biliverdin and bilirubin glucuronide in living organisms and experimental models. This approach was aimed to avoid other methods that require the solvent-extraction of bilirubin and generate hazardous waste, like HPLC and LC-MS methods (e.g., Ref. [4]), and that require advanced laboratory equipment and a trained workforce. We addressed the need to avail of a simple, cheap and high-throughput assay, accessible to basic laboratories operating in pre-clinical or clinical research, requiring interference-free measurements. On these premises, we decided to employ UnaG for the fluorometric analysis of bilirubin, producing it as a recombinant fusion with the HELP carrier in bacteria and purifying it by exploiting the thermoresponsive properties of HELP.

Bilirubin is a yellow pigment found in animal plasma. Bilirubinemia is one of the most frequent blood tests undertaken to check the functional status of the liver [5], and values above 1 mg/dL are indicative of liver dysfunction and require further diagnostics [6]. Analyses are performed by an automated colorimetric method that measures the sum of bilirubin and its hepatic metabolite bilirubin glucuronide and, separately, bilirubin glucuronide, so that bilirubin concentrations are obtained by calculus. Remarkably, the latter fraction, also known as indirect bilirubin, is >90% under normal conditions. In the last decade, mild elevations of bilirubinemia that raise no disease concern have been found to be associated with a decreased disease risk [7], triggering strong interest in understanding the clinical relevance of this finding and in undertaking research to understand the factors that cause fine modulation of bilirubin metabolism and disposition. To this purpose, the use of pre-clinical experimental models is mandatory, but, in this case, the automated colorimetric method used in clinical chemistry cannot be applied to samples other than plasma. In several pre-clinical investigations, only HPLC-based methods could be used to isolate and quantify bilirubin and bilirubin glucuronide. Indeed, unlike the HPLC methods, the colorimetric diazo method is afflicted by interferences that undermine the quality of results in the normal range [8].

The aim of this review is to present the development of HUG from its conception to the implementation and application in different experimental models and research questions. HUG represents a case study of upward progress on the scale of technology readiness levels (TRL), a metric tool adopted by large R&D organizations or funding agencies to communicate the extent to which a given technology is scientifically validated and reliably applicable to the real world, i.e., outside the research laboratory [9,10]. There are nine TRL stages, spanning from the initial idea and outlining its theoretical background (TRL 1) to the description of the technology features and the experimental plan to create it (TRL 2). The first experimental demonstration and validation in a laboratory correspond to TRLs 3 and 4. The demonstration of the technology performance in a real environment(s) achieves TRL 5, and the creation of its pre-industrial prototype corresponds to TRL 6. TRLs 7 and 8 are associated with technology maturation at the industrial level (scaling-up, production process, large-scale technology applications, regulatory approval). TRL 9 is the stage of marketing. The wording and definitions are not rigid and can be adapted to specific science and technology domains, though the fundamental level distinctions are common, making the TRL scale a powerful tool to facilitate communication and management in innovation ecosystems [9].

**Figure 1:**
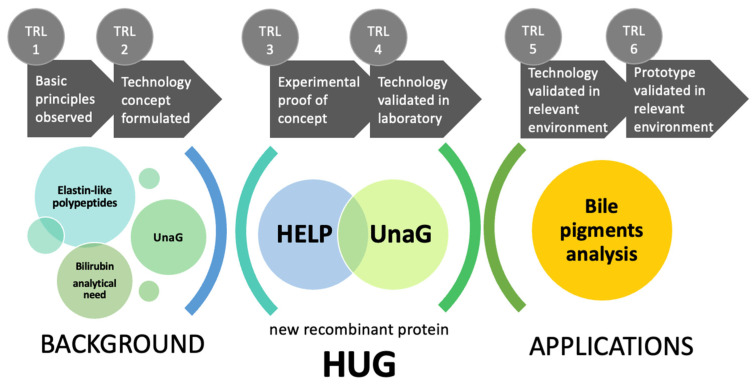
The technological development of the HELP-UnaG fusion protein HUG.

Here, we present the technological development of HUG from its initial idea and its scientific background (TRL 1) to the current stage of a prototype (TRL 6) ready to be taken up and possibly further developed to full maturity (TRL 7–9) (Figure 1).

## 2. Basic Principles and Technology to Produce HELP-UnaG

The aim of creating a UnaG fusion product with the HELP carrier was based on our experience in producing recombinant HELP fusion products, such as HELPc [11], HELP Epidermal Growth Factor (HEGF) and HELP-RGD, which promoted cultured muscle cell differentiation [12]. One of the most attractive features of HELP fusion proteins is that they retain to some extent the thermoresponsive behavior known as the inverse thermal transition that can be exploited for their purifications.

The theoretical foundations required for the successful development of new recombinant HELP fusion proteins such as HUG are described below. They were not only the basis for the design, cloning, expression and purification of this new HELP fusion protein but were also the guide for further improvements and technological advancements. This description covers the first three levels of the TRL scale.

### 2.1. Engineered Elastin-like Polypeptides

Elastin is one of the main components of the extracellular matrix and one of the most studied structural proteins. It is characterized by rubbery elasticity and is a functional component of tissues such as blood vessels, skin and lungs [13]. Its precursor, tropoelastin, is rich in hydrophobic Val-Pro-Gly-Val-Gly (VPGVG) motifs and exhibits phase transition behavior at a low critical solution temperature (LCST). Tropoelastin is water-soluble below its transition temperature (T_t_), whereas above it, the protein aggregates and separates as a second phase [14]. This peculiarity has inspired the study of engineered elastin-like polypeptides (ELPs), derived from the hydrophobic domain of bovine tropoelastin and retaining coacervation properties [15]. Like tropoelastin, ELP solutions also display LCST phase transition behavior [16,17,18]. This phenomenon, also known as the inverse thermal transition (ITT), is a phase transition of an ELP solution occurring at a specific solution temperature, T_t_, which drives the formation of both intra- and intermolecular hydrophobic interactions, resulting in protein folding change and assembly [19]. Above the T_t_, intermolecular polypeptide interactions are favored over polypeptide−solvent interactions, so that the protein separates from the solution. In this process, the regularly arranged water molecules of the hydrophobic hydration shell around ELPs become less ordered molecules, as in unperturbed bulk water [20], leading to a net increase in the entropy of the system.

ELPs are designed by a modular approach using variations of the VPGXG building block, where X can be any guest amino acid, except for proline, because of its chain conformation-disrupting properties [21], and the number and type(s) of repeated motifs can vary [22,23,24]. This paves the way for producing versatile ELP-based biomaterials [25], with fine-tuned hydrophobic indexes, thermosensitivity and gel-forming properties.

A sub-class of ELPs is given by engineered human elastin-like polypeptides (HELPs), featured by the hexapeptide repetitive domain Val-Ala-Pro-Gly-Val-Gly (VAPGVG) and, different from most of the other described ELPs, by the cross-linking domains found in human tropoelastin [26].

### 2.2. Design and Cloning of HELP in E. coli

**Figure 2:**
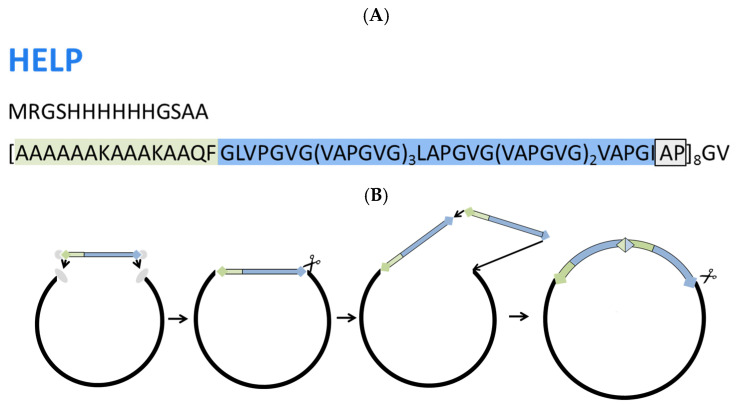
(**A**) Primary structure of the recombinant eight-block human elastin-like polypeptide. The gray box indicates the possible point of in-frame insertion of any domain. (**B**) Schematic representation of the RDL-like cloning strategy followed to obtain the multimerization of the human elastin-derived module. The first module was cloned between two different unique restriction sites of the plasmid. The cross-linking and elastin-like hydrophobic domains are highlighted in green and blue, respectively.

HELP (MW = 44,886 Da) was designed to express two functional domains encoded by exons 23 and 24 of the human tropoelastin gene. The alanine-lysine-rich cross-linking domain, encoded by exon 23, is followed by the hexapeptide VAPGVG, encoded by exon 24 [26]. This bifunctional unit is repeated eight times [27] (Figure 2A). The sequence comprises a His-tag for exploiting affinity-based recognition by monoclonal antibodies. In addition, it was observed that the His-tagged HELP was expressed at higher levels with respect to the untagged HELP, resulting in an improved yield. The pEX8EL plasmid was used.

The synthetic HELP gene was cloned into a T5 promoter-based expression vector so that the inducible expression could be achieved by a lac*Iq* strain (Figure 2B). The HELP expression construct was co-transformed into the NEBexpressIq *E. coli* strain. The conditions of the optimized HELP expression system are reported in Table 1.

### 2.3. Purification of HELPs

The scalability and standardization of the purification process determine the potential industrial production of elastin-like engineered proteins [28,29].

One of the possible methods for the purification of ELPs is the use of a His tag for immobilized metal affinity chromatography (IMAC) [30]. However, this method has some limitations and difficulties in scaling up, such as high costs due to the use of resins and the need for hazardous reagents (e.g., imidazole as a competitor molecule).

Non-chromatographic ELP purification methods like the organic extraction from whole cells and cell lysates with negligible contamination by nucleic acids or lipopolysaccharides are rare [31,32,33,34,35].

The preferred method for purifying ELPs takes advantage of their unique phase transition behavior, which achieves purification by inverse transition cycling (ITC) [36,37,38,39]. This simple and efficient method consists of hot centrifugation in a high ionic strength solution leading to a protein pellet made of ELP, followed by the pellet resuspension in cold water. The latter is centrifuged at low temperature to remove impurities. This hot–cold cycle can be repeated to obtain a pure product, though at the detriment of yield. Overall, the ITC method provides comparable or better results than the IMAC method, as reported by Ref. [40], showing that the ELP-conjugated superoxide dismutase (ELP-SOD) purified by ITC was equivalent or even better than that purified by Ni-NTA resin and ion exchange chromatography. With ITC, a scale-up from micrograms to milligrams is possible, and the technique can be optimized for high-throughput purification.

### 2.4. Physico–Chemical Features of HELP

Knowledge of the macromolecular parameters of HELP is the rational basis for establishing an efficient ITC-based purification protocol from bacterial extracts [14,26,41,42,43]. In addition, HELP is the reference polymer for the macromolecular characterization of new HELP fusion proteins and for the optimization of their purification and further technological utilization. We have described the aggregation properties and thermoresponsive behavior of the biopolymer HELP under different conditions using turbidimetry, differential scanning calorimetry, circular dichroism and dynamic light scattering [2,44,45].

The ability of HELP to undergo an inverse temperature transition under certain solution conditions, mainly at low and near-physiological salt concentrations, was determined by turbidimetric measurements. Under these conditions, HELP showed an inverse transition process very similar to that described for other elastin-like polypeptides [46,47]. Table 2 shows the T_t_ for HELP at different solvent conditions [45,48,49,50]. The HELP T_t_ was similar in different solvents, such as PBS, Tris and sodium phosphate buffer (NaPi), even with pH changes from 7.3 to 8.0. The presence of physiological salt concentrations (0.15 M NaCl) resulted in a marked effect on T_t_ and, remarkably, in an efficient coacervation process, which was evident in the form of a turbidity profile [2]. In contrast to other ELPs, where there are no cross-linking domains, the presence of a near-physiological NaCl concentration is essential to achieving or promoting the coacervation process of HELP and HELP-based fusions.

Differential scanning calorimetry (DSC) was used to characterize the inverse phase transitions of HELP in the presence of different NaCl concentrations [45]. A single endothermic peak with an asymmetric shape was recorded from which the T_t_ transition enthalpies (ΔH_tr_) and entropies (ΔS_tr_) were determined (Table 3). In the entire salt concentration range (0.1–0.9 M NaCl), an entropy-driven process of dehydration was observed, as previously described for other elastin-like polypeptides [43,47]. Within this salt concentration range, HELP showed a constant linear increase in both transition enthalpy and entropy [2], as described by other authors for different ELPs [47], confirming that the presence of the cross-linking domains must be “neutralized” by at least one physiological salt concentration, making it an ELP. A very important result is that the low hydrophobicity of the HELP protein is associated with low values for the transition of energy and entropy, as generally observed for ELP polypeptides [50,51].

When different solvents were considered, i.e., Tris and NaPi as mixed solvents, no remarkable differences were found between the T_t_ values in the absence of NaCl salt (29–29.5 °C) (Table 3), which agrees well with the values obtained by turbidimetric techniques. The addition of the salt to the HELP solutions, responsible for shielding the charges and avoiding chain repulsions led to an increase in T_t_ (34–39 °C), with little differences between the different mixed solvents used.

Looking at the enthalpic and entropic results of the DSC (Table 3), it becomes clear that the effect of the ionic strength, destabilizing the water structure, results in less water available for the formation of cage-like structures around non-polar protein groups than in the lattice of unperturbed water. Thus, a remarkable entropy change was measured during the inverse transition process due to the loss of a relevant number of water molecules from the hydration structure, and a significant decrease in entropies was observed when NaCl salt was added to the solutions (Table 3).

Furthermore, significant differences were found between the enthalpies measured in different solvents, the values of which are related to the interactions between non-polar groups, ions and unperturbed water in the extended hydrophobic sphere and to the specific and different types of ordering in the local water (Table 3).

Circular dichroism (CD) spectra at different temperatures in aqueous and salt solutions were recorded [2,45]. The results showed the typical, well-documented CD profile of elastin and the short ELP polypeptides [16,52], which is characterized by a high percentage of random coil structure with a negative band around 200 nm (ππ* transition). The negative peak at 222 nm (nπ* transition) [16,53] was related to both α-helical segments (222 nm) and type I/type II β-turn and PP-II secondary structures at 225 nm. After the deconvolution of the CD spectra, the HELP sequence resembled the distribution of secondary structures predicted for human and bovine elastin, with a proportion of 20–29% α-helical domains, 60–63% of random coil and about 10% of β turn structures [48]. This pattern is typical of those of human and bovine elastin and similar to other short ELPs [2]. The secondary structure prediction for the HELP protein was calculated as the sum of the hydropathy values of all amino acids normalized by the number of amino acids in the sequence by the ProtParam (Expasy) program [54], which uses the GOR IV method to predict the secondary structure from the primary amino acid sequence [55]. The predicted results were consistent with the experimental data, resulting in an average value of 28% of α-helix content, about 70% of random coil and a low percentage of β structures, pointing to a prevalent disordered structure for the HELP protein.

Dynamic light scattering (DLS) was used to determine the hydrodynamic radius (R_H_) of HELP in a solution, as well as the dimensions of aggregates at different temperatures and concentrations. From the Stokes−Einstein theory, the diffusion coefficients D and then the hydrodynamic radius R_H_ were calculated [56]. The percentage of the peak areas was obtained from intensity, volume and number distributions by nonlinear least-squares fitting.

Two modal size distributions were observed in the temperature range of 10−45 °C with an average R_H_ value of 50 Å (78%) between 10 and 30 °C, which increases to about 1000 Å when the temperature rises to 45 °C due to the onset of the aggregation process [45]. If the temperature is raised above the T_t_ value, a complete aggregation process with a single modal distribution occurs, in which particles with an average R_H_ value of more than 1000 Å are formed.

### 2.5. HELP Recombinant Fusion Proteins

Recombinant HELP fusion proteins retain the phase transition property of HELP. In this way, several recombinant HELP fusion proteins can be purified by the same ITC protocol, with minor modifications [37,57,58]. However, the feasibility of ITC-based purification must be assessed on a case-by-case basis to rule out any interference by the fused domain.

It was observed that all the HELP fusion proteins produced up to now were expressed in soluble form without the formation of inclusion bodies, as previously described for HELP [26]. Among the other advantages, the enhancement of protein expression was frequently observed as well as the retaining of the thermoresponsive behavior that allows for performing ITC-based purification. In addition, linkers and specific proteolytic sites can be inserted between HELP and the fusion domain for the release of this last (for example, see Ref. [49]).

Several bioactive ELPs as well as HELP fusion proteins have been developed for multiple purposes. The fused functional domain Arg-Gly-Asp (RGD) or the Epidermal Growth Factor (EGF) [12,59,60] have been exploited in in vitro cell cultures to enhance cell adhesion and differentiation [61,62,63,64]. Antimicrobial peptide fusions are used to develop bioengineered drugs [65,66].

On these premises, the design and production of a recombinant HELP-UnaG fusion was considered a feasible option. UnaG is produced by recombinant DNA technology as fusion products with histidine tails or proteins such as glutathione transferase [67], or maltose-binding proteins [68]. These adducts are ultimately enzymatically cleaved and removed by further chromatographic steps to obtain pure UnaG. We preferred to express a HELP-UnaG fusion product to obtain a new bi-functional polypeptide that may retain the properties of both carrier and fusion domains [1].

### 2.6. UnaG

UnaG is the first protein that can become fluorescent upon ligand binding obtained from a vertebrate. Its non-covalent binding to bilirubin, its physiological ligand, results in a green fluorescent complex. UnaG was first cloned and described in 2013, being isolated from the muscle fibers of the freshwater Japanese eel (Unagi, or *Anguilla japonica*) [3]. UnaG consists of a beta-barrel structure determined by 10 antiparallel beta-strands. In holoUnaG, the bilirubin is in the center of the cavity formed by the beta-barrel. As can be seen from the atomic structure of holoUnaG, the high binding affinity between bilirubin and UnaG is determined by numerous interactions based on the formation of hydrogen bonds with appropriately and orderly positioned water molecules and amino acid residues [3].

It belongs to the family of fatty acid-binding proteins, binds bilirubin with high affinity and specificity and produces fluorescence even under anoxic conditions [69].

Since its discovery, UnaG has attracted the attention of the scientific world due to its potential for clinical and technological applications in the preclinical field.

The high affinity (*K*_d_ = 98 pM) of the protein to its ligand makes it an excellent tool for direct dosing of unconjugated bilirubin even in blood samples. The fluorescence emission is not affected by the presence of other molecules such as hemoglobin, and thus sample processing takes less time. In addition, the UnaG binding with bilirubin is much stronger than the binding of bilirubin with albumin, so no deproteinization of the plasma is required either [3].

The expression of UnaG has been performed and optimized both in bacteria, to employ the purified apoprotein for clinical applications, and in eukaryotic cells, for technological applications, to study cellular heme metabolism or as a reporter (Table 4). In bacteria, the apo-protein (MW = 15,581 Da) is expressed with a tag that allows for affinity purification. The different uses and related purification methods are presented in Table 4.

## 3. HUG Assay: Features and Use for the Fluorometric Analysis of Bile Pigments

We tested and validated all parameters of the HUG and of the assay based on this tool in five distinct projects, culminating with the publication of research and methodology articles. First, we performed the physico–chemical characterization of HUG for both its general macromolecular properties and as a specific bilirubin-dependent fluorophore [45]. Then, we standardized its lab-scale production [124]. We established a procedure for preparing quality-controlled bilirubin standard solutions spanning the range of 10^−3^–10^−9^ M [125]. We set up a method for the nanoscale analysis of bilirubin by HUG that yields results matching the standard bilirubin analysis in clinical chemistry [126], and we upgraded it for the combined analysis of both biliverdin and bilirubin in the same sample [127]. In another project, we included the validated analysis of bilirubin glucuronide by upgrading the HUG assay with the enzyme β-glucuronidase [128].

### 3.1. Design and Cloning of HUG in E. coli

**Figure 3:**
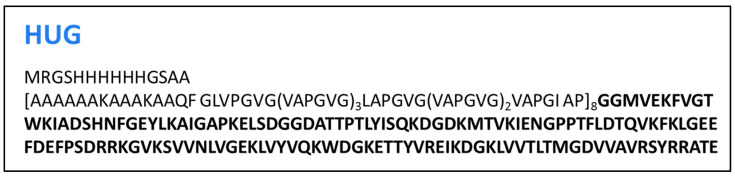
Primary structure of the chimeric HUG polypeptide. In bold is reported the amino acid sequence of UnaG. Theoretical molecular weight is 60,406 Da.

The engineered HELP protein was fused with the UnaG eel protein to obtain a chimeric polypeptide, which was named HUG [1]. For the realization of HUG, the recombinant gene of the HELP polypeptide was fused with the 139 amino acid-coding sequence of the UnaG bilirubin-binding protein (accession number BAN57322.1; GenBank), exploiting the unique *DraIII* site in the expression vector that allows for the in-frame insertion of the polypeptide at the C terminus (Figure 3).

The fusion product was expressed in the C3037 *E. coli* strain, and the conditions of the optimized HUG expression system are the same as those reported in Table 1.

### 3.2. The Protocol to Prepare Standardized Lots of HUG

**Figure 4:**
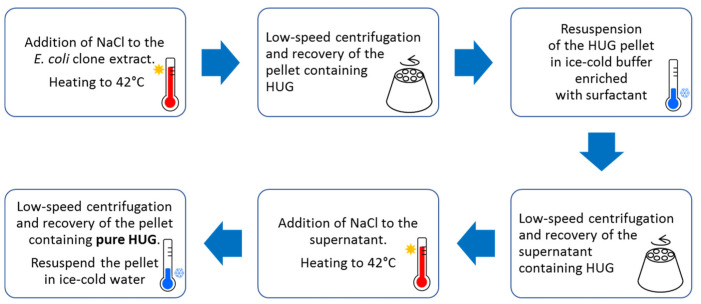
Sequential steps of the cycle for purification of HUG.

The HUG recombinant protein was produced and purified as reported [124]. The protocol for the purification of HUG from induced *E. coli* biomass extracts is based on the addition of NaCl to the soluble fraction of the extract to lower the inverse transition temperature and promote the coacervation process. Large HUG aggregates are sedimented by centrifugation at low speed (<10,000 rpm). The resulting pellet is redissolved by cooling. This temperature-dependent transition from the liquid to solid phases is repeated several times to obtain the purified HUG protein (Figure 4). The purification protocol differs slightly from that intended for HELP since it requires the use of sodium deoxytaurocholate to remove lipid residues that the fatty acid-binding protein UnaG may have co-precipitated.

The final product is subjected to quality control to evaluate purity, concentration and bilirubin-dependent-specific fluorescence. Under these standardized conditions, the HUG exhibits a specific UV–Vis extinction coefficient ε_280_ = 18.747 and a bilirubin-dependent-specific fluorescence of 11.663 A.U./μg. The protein yield averages 200 mg per liter of bacterial culture. The production of 0.5 g HUG requires 5 days of experimental work when the bacterial clone is already available [124]. Considering that the standard protocol for nanoscale fluorometric analysis of bilirubin requires 1 mg of HUG for a 96-well plate suitable for the analysis of 24 samples (among which the calibration standards), each in quadruplicate, a lot of 1 g of HUG obtained from 5 L of bacterial culture enables the analysis of 24,000 samples.

### 3.3. Physico–Chemical Features of HUG

The macromolecular characterization of HUG [45] has provided insights into the solution properties of the protein chain and into its capacity to form a complex with bilirubin. First, as in the case of the HELP polypeptide, thermodynamic and spectroscopic techniques were used to analyze the reverse-transition process of the HUG. In addition, the binding capacity of the HUG was investigated under various solution conditions using fluorometric methods.

The features of the HUG macromolecule were predicted using the program ProtParam (Expasy) based on the primary structure of the protein. With respect to the HELP biopolymer, the secondary structure distribution for HUG showed a greater proportion of β-strand conformation (10%) due to the UnaG contribution. These calculations showed, in addition, that both sequences of HELP and UnaG retain their secondary structure distribution in the HUG fusion protein.

**Figure 5:**
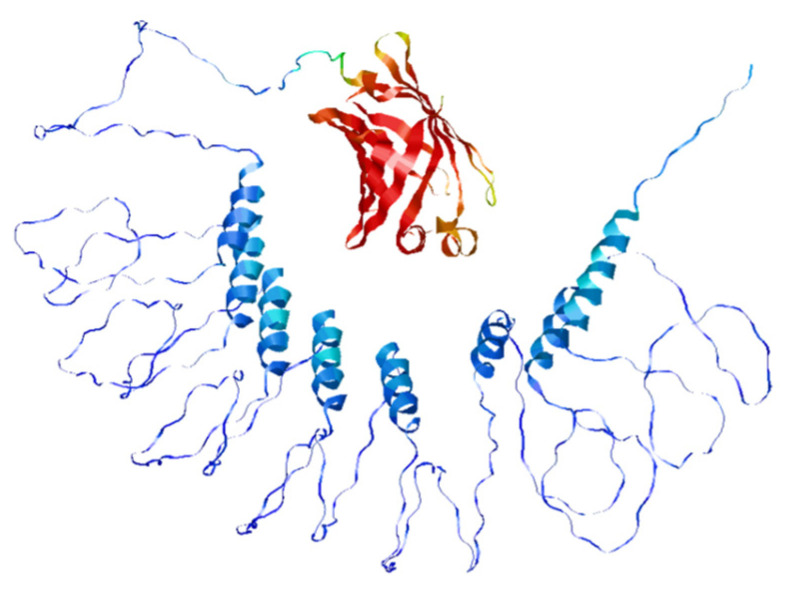
Model of the tertiary structure of HUG. The model is an AlfaFold2 minimized structure of HUG, with the UnaG domain (red ribbon) connected to the HELP biopolymer (blue ribbon).

The CD spectra of the HUG solutions showed the typical shape previously observed for HELP biopolymers [2] and generally shared by the ELP polypeptides [16,53]. Less pronounced negative bands at 200 nm and 222 nm for HUG compared to HELP were recorded due to the changes in secondary structure distributions with a lower proportion of α-helix and random coil sequences in the HUG polypeptide [45]. Accordingly, molecular modeling performed with AlfaFold2 in vacuo [129] returned the tertiary structure, as shown in Figure 5.

The inverse thermal transition of HUG was investigated using turbidimetry and DSC techniques. As shown for HELP, the HUG biopolymer underwent the typical hydrophobic process as a function of temperature (coacervation) with a strong increase in turbidity. Under different solvent conditions (PBS and Tris/NaCl), HUG showed little change in T_t_ compared to HELP solutions, with a value in the range of 31.9–33 °C [45].

The entropic and enthalpic contributions were determined from the DSC measurement. The lower chain hydrophobicity calculated for HUG compared to HELP resulted in a significant decrease in ΔH_tr_ of HUG (in PBS, pH = 7.4), mainly due to the different charged groups present, while the similar number of water molecules in the solvation spheres made a similar contribution to ΔS_tr_ [45].

Using the DLS technique, the dimensions of the HUG aggregates of different sizes at different temperatures and concentrations were measured. With an average particle radius of 62, 250 and >150 Å, a three-modal size distribution was observed as a function of temperature [45].

The fluorescence spectral characteristic of the complexes of bilirubin with UnaG and HUG is the same, as indicated by the peak values derived from their excitation and emission spectra, with excitation maxima at 498 nm for both UnaG and HUG and 527 or 530 nm for UnaG and HUG, respectively [3], [1]. A refined estimate of the binding of UnaG with bilirubin revealed that the K_d_ of the complex is 0.031–0.098 nM [3,67] which results from the parameter of a simple hyperbola. Although the K_d_ value of the HUG–BR complex (1.1–1.7 nM) [1,45] is two-to-three orders of magnitude higher than that of UnaG-BR, it is still lower than that of the albumin–bilirubin complex (45 nM) [130]. Therefore, the HUG can efficiently displace Br from BSA and enable the determination of bilirubin even in biofluids in which bilirubin forms a complex with serum albumin (typically in blood).

### 3.4. The Protocol to Prepare Standard Solutions of Bilirubin

Methods that analyze bilirubin aqueous solutions in the nanomolar scale encounter the challenge of calibration, which impacts the assay performance, reliability and comparison of results across different methods and real samples. Bilirubin is a lipophilic compound with an upper limit of solubility in water <100 nM and a marked instability. Bilirubin standards in the nanomolar range are not commercially available. Moreover, we found quite scant published information on how bilirubin standards are prepared in different methods. We addressed this gap of knowledge by establishing a standardized protocol for the preparation of quality-controlled standard bilirubin solutions [125]. To overcome chemical instability, we supplemented standards with low amounts of bovine serum albumin, which does not interfere with the assay because the affinity HUG for bilirubin is higher than for albumin(s), and therefore any albumin-bound bilirubin molecule will be transferred to HUG at equilibrium.

### 3.5. The HUG-Based Method for the Nanoscale Analysis of Bilirubin

**Figure 6:**
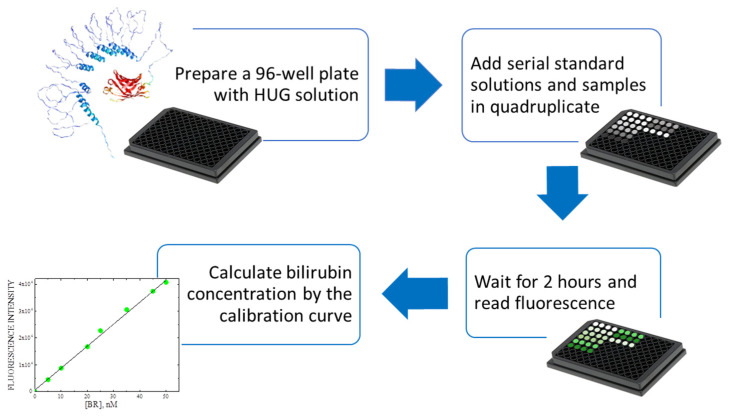
Workflow of the HUG assay. The 96-well plate is filled with standard solutions and samples (grey scale colors). After 2 h, steady-state green fluorescence (green scale colors) is recorded to calculate bilirubin concentration.

The HUG method for the nanoscale analysis of bilirubin [126] exploits the 96-well plate configuration, as described in Figure 6. A HUG solution is distributed in the plate’s wells. Standard solutions of bilirubin are added at concentrations 0–50 nM to obtain a calibration curve. The samples are distributed in the wells and the emitted fluorescence is quantified by a microplate reader.

The validation parameters of the method are shown in Table 5.

After extracting information from a recent review that compares 42 methods for the analysis of bilirubin [131], in terms of LOQ, the HUG-based method ranks third among 27 fluorescence methods and is comparable to the best HPLC method coupled to MS, but it is outperformed by three electrochemical methods based on nanomaterials. However, a crucial feature of the HUG assay is that it does not require sample preparation, advanced equipment or specialized technical skills.

The robustness of the method was assessed with five common buffered solutions. The angular coefficients (nM^−1^) of the calibration curves were 551–663. The supplementation of phosphate-buffered saline solution (PBS) with bovine serum albumin led to an increased angular coefficient (785 nM^−1^), whereas the addition of the surfactant Tween 20 at 1% produced an angular coefficient of 7.5 nM^−1^, likely due to incompatibility with HUG. Other surfactants, such as 1% Triton X-100 or 0.2 mM sodium taurocholate, had no major impact on this parameter. Quite remarkably, DMSO up to 30% was tolerated, which is a major advantage when assessing bilirubin in the presence of other lipophilic compounds.

In another study, we demonstrated further details about the robustness and specificity of the method, showing that a series of drug molecules used to inhibit the hepatic uptake of bilirubin did not interfere with the assay, i.e., estradiol 17-β-glucuronide, pravastatin, ketoprofen, cyanidin 3-glucoside, indomethacin and taurocholate [128].

When applied to human plasma, the precision of this method (Table 5) enables us to discriminate the bilirubin phenotype pattern linked to genetic polymorphisms of the gene *UGT1A1*, which determines graded mean increases of conjugated bilirubin in the blood. Similarly, the method enables us to detect well-known sex-related differences in bilirubinemia [126].

### 3.6. The Upgraded HUG Method for the Analysis of Biliverdin and Bilirubin Glucuronide

The configuration of the HUG assay in 96-well plates enables us to prepare wells not only with HUG but also with HUG plus biliverdin reductase and NADPH to obtain the total conversion of biliverdin to bilirubin [127]. Similarly, wells can be prepared with HUG and β-glucuronidase that catalyze the hydrolysis of bilirubin glucuronide(s) to bilirubin [128]. The presence of HUG in the reaction wells traps the main reaction product bilirubin and therefore drives both reactions to completion. Appropriate controls must be included to pinpoint unwanted fluorescence artifacts.

### 3.7. The Technological Readiness Level of the HUG Assay

**Figure 7:**
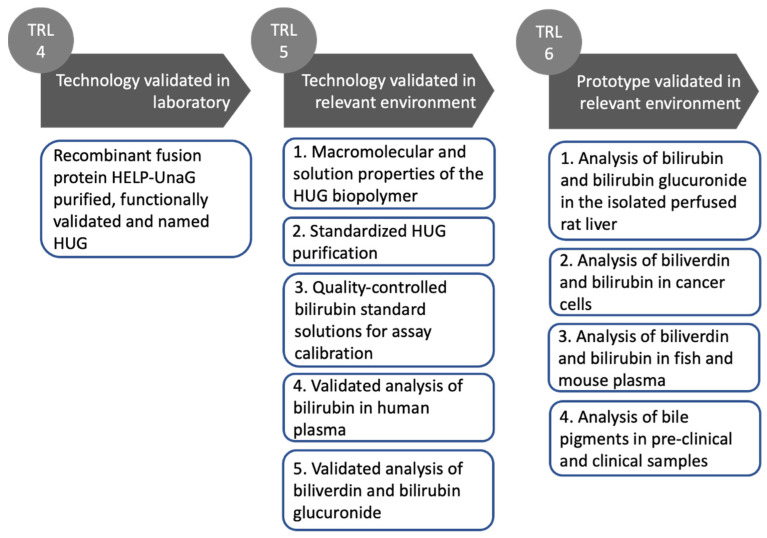
Progression through the technology readiness levels of HUG and its use in the fluorometric assay of bilirubin, biliverdin and bilirubin glucuronide. TRL 4 was achieved by producing the new HELP-UnaG fusion protein, retaining the main functions of its individual protein domains [1]. TRL 5 was achieved by characterizing the components and processes of the HUG assay: 1. The structure and function of HUG [45], 2. The HUG purification protocol [124], 3. The preparation of bilirubin standard solutions [125], 4. The HUG assay of bilirubin in human plasma [126], and the combined analysis of bilirubin and biliverdin [127]. TRL 6 was achieved by applying the HUG assay to quantify bilirubin and/or bilirubin glucuronide and/or biliverdin in biological samples, such as: 1. The effluent of the isolated perfused rat liver [128], 2. Metastatic cancer cells grown in vitro [132], 3. The plasma collected from farmed fish or biliverdin reductase -/- C57Bl/6 mice [127], 4. Human plasma biobanks and rat blood (work in progress).

The scheme presented in Figure 7 provides an overview of the experimental steps undertaken to characterize the performance of the HUG assay, framing them in the technology readiness levels paradigm.

## 4. Bile Pigment Analysis in Biology and Medicine: Theory and Applications

**Figure 8:**
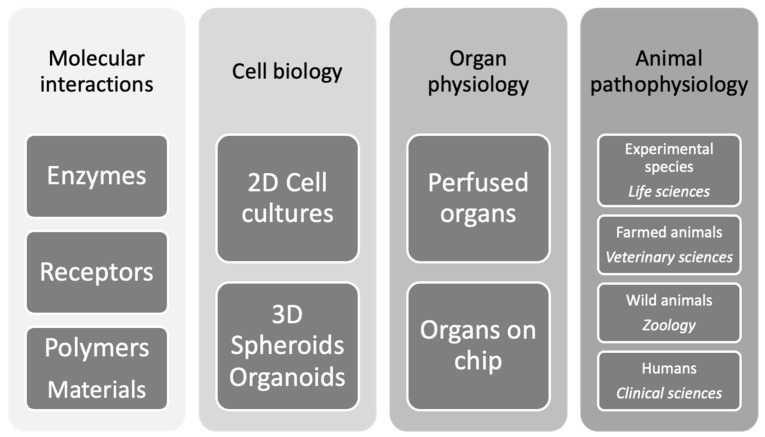
Spectrum of applicability of the HUG assay in biology and medicine.

The HUG assay opens the possibility for any laboratory to use the same method for advanced bile pigment analysis spanning a wide arch of biomedical translational research and reaching clinical studies (Figure 8).

This is a great advantage for a research group since a given hypothesis can be tested in experimental models of different degrees of complexity (e.g., isolated proteins or cells; micro-physiological systems like organoids, experimental animals and so on) by availing of the same analytical approach. As a result, not only can the reproducibility of the results be repeatedly tested and confirmed but even more new biological principles may emerge. Indeed, we anticipate the improvement of our still imprecise understanding of the fine regulation of the production of bile pigments from heme catabolism and hepatic bilirubin elimination. The biology of these processes is summarized below.

### 4.1. Sources of Heme

Heme plays vital roles in aerobic life, such as the binding of oxygen for cellular storage (myoglobin), scavenging reactive oxygen species (catalase, peroxidase), electron transport (cytochromes), synthesis of bioactive molecules (cyclooxygenase, nitric oxide synthase), regulation of gene expression (heme response elements) and transport of oxygen to the tissues via blood circulation (hemoglobin). Many cellular functions are influenced by the rate of heme catabolism, such as cellular oxidative stress response, inflammation and related diseases of the central and peripheral nervous system, cardiovascular and respiratory systems and cancer [133]. Since bilirubin is eliminated by biliary excretion after hepatic metabolism, bilirubin is a primary biomarker of liver disease. The largest pool of heme (about 75%) is found in hemoglobin and is released in erythrocyte turnover in the spleen. Accordingly, hemolytic diseases produce large amounts of bilirubin. The remaining fraction of heme derives from the ubiquitous cellular catabolism of heme-bound enzymes [134], a process that is regulated by a multitude of stress factors.

### 4.2. Synthesis of Bile Pigments

**Figure 9:**
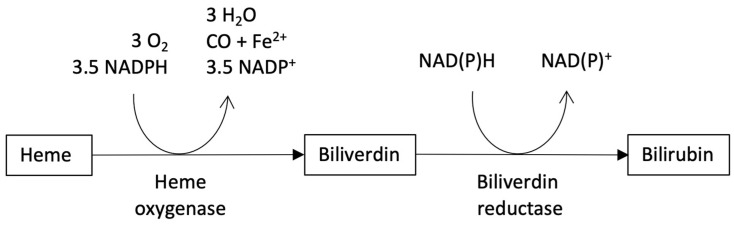
Biosynthesis of bile pigments. Heme is cleaved in a NADPH+H^+^-dependent reaction catalyzed by heme oxygenase, whose products are biliverdin, CO and Fe^2+^. Bilirubin is the product of the reaction catalyzed by NAD(P)H+H^+^-dependent biliverdin reductase. The reaction scheme is in accord with Ref. [135].

Both biliverdin and bilirubin are tetrapyrrolic molecules synthesized in animal cells from heme in a two-step reaction pathway, catalyzed by heme oxygenase (HO) (EC: 1.14.14.18) and biliverdin reductase (E.C. 1.3.1.24) (Figure 9). The products of HO are biliverdin, carbon monoxide and ferric ion in equimolar stoichiometry. The diffusion of CO in the body and the atmosphere drives the HO reaction to completion. In mammals, the reaction of biliverdin reductase is essentially irreversible, with a complete conversion of hydrophilic biliverdin to lipophilic bilirubin [135]. This may be ascribed to the fact that bilirubin diffuses from the cells into the blood, where it forms a high-affinity complex with serum albumin (45 nM) [130], leaving the unbound fraction to nM concentrations [136].

HO is expressed in non-erythroid tissues in two isoforms, HO-1 and HO-2, encoded by two genes [135,137], which catalyze the same reaction though with different enzyme kinetics parameters. The former is inducible under a variety of cellular stress conditions, whereas HO-2 is seen as the isoform that covers the basal needs of heme turnover [137]. HO-1 induction plays a major role in cytoprotection, observed at all levels of complexity from in vitro experimental models to the beneficial effects of all major *apparatus* of the human organism [138]. An excessive induction of HO-1 can, however, be detrimental, mostly because of the excessive production of free iron [139]. Indeed, some anticancer drugs do act by abnormally increasing the expression and activity of HO, leading to desired cell death. By using HUG for the combined analysis of biliverdin and bilirubin, we could demonstrate that the upregulation of HO mRNA and proteins was paralleled with a significant increase of intracellular biliverdin in the metastatic cancer cell line [139]. There is a huge research interest in understanding the regulation of HO and the long-range impact of changes in its expression, as indicated by >16,000 items in PubMed. Adding the analysis of biliverdin in cells, tissues and biofluids to the current practice of quantifying HO mRNA or proteins provides a deeper phenotypic understanding of this metabolic hot point.

### 4.3. Bilirubin Elimination

Bilirubin is selectively taken up into the liver by a mechanism that is still poorly characterized, with questionable involvement of sinusoidal membrane transporters known as organic anion transporting polypeptide 1B1 (OATP1B1, *SLCO1B1*) and OATP1B3 (*SLCO1B3*) [140]. The likely involvement of other membrane transporters has been demonstrated by analyzing both bilirubin and bilirubin glucuronide in the outflow of the isolated perfused rat liver by a HUG assay [128]. In the liver, bilirubin is metabolized to bilirubin mono- and di-glucuronide by the specific UDP-glucuronosyltransferase UGT1A1 (EC 2.4.1.17) and excreted into the bile by the biliary ATP-dependent transporter MRP2 (*ABCC2*) [134,140].

### 4.4. The Bioactivity of Bile Pigments

#### 4.4.1. Biliverdin

Biliverdin is the direct conversion product of heme catalyzed by a family of enzymes known as heme oxygenase. In the cytoplasm of the cell, biliverdin is normally converted to bilirubin by biliverdin reductase and subsequently excreted into the plasma or extracellular environment.

Biliverdin is a more hydrophilic compound than bilirubin and is non-toxic; moreover, it can be excreted as such in both bile and urine [141]. Together with bilirubin, it is known for its antioxidant, anti-inflammatory and anti-apoptotic effects.

The antioxidant action of biliverdin may protect the brain from ischemia-reperfusion injury via the Nrf2/A20/eEF1A2 axis and inhibition of pyroptosis [142]. Antioxidant activity was also detected in the erythrocytes of 9-month-old Bvra-/- mice, in which oxidative stress was significantly reduced [143]. In addition, the antioxidant activity was associated with the BV/BR cycle in cells, and thus with the activity of enzymes involved in this process, such as heme oxygenase and biliverdin reductase, which can reduce the apoptotic activity of cisplatin in NRK-52E renal cells [144]. It has also been shown that this molecule can act as an antitumor molecule via a mechanism related to its antioxidant activity by modulating the angiogenic pathway [144]. The anti-inflammatory effect of BV is due to its ability to induce and bind BVR present on the macrophage membrane, which in turn activates the PI3K–Akt signaling pathway that triggers the anti-inflammatory response [145].

#### 4.4.2. Bilirubin: Observational Studies

In the absence of hemolytic anemias or liver diseases that cause different patterns of hyperbilirubinemia and jaundice in adult life, mild elevations of total bilirubinemia within the physiological range are associated with the decreased risk of several disease conditions and different populations. The first observation of a negative correlation between cardiovascular diseases and total bilirubinemia was reported in 1994 [146]. Several other clinical studies followed that investigated correlations with a wider disease spectrum. A series of metanalyses established that the participant groups with the highest bilirubin levels had a significant disease risk. For example, data from 12 prospective studies involving 368,567 participants identified a decreased cardiovascular disease risk (by 25%) [147]; data from 11 cohort studies involving 263,596 participants identified a decreased stroke risk (by 15%) [148]; and data from 10 studies involving 79,508 individuals found a decreased risk of metabolic syndrome (by 30%) and type 2 diabetes (by 22%) [149].

Of related interest is also the observation of a significant negative correlation between total bilirubin and the systemic immune–inflammation index that reflects the balance between inflammation and immune response [150].

When available, for instance in Refs. [147,150,151], the dose–response curve showed the strongest decrease of disease risk occurred in the hypobilirubinemic range, i.e., <10 µM, taken as the reference normal value. Given that subtle changes of bilirubinemia at values <10 µM are related to sizeable changes in the disease risk [7,152], it is evident that accurate measurements of total bilirubinemia and its fractions are necessary for correct understanding of study results.

Similar beneficial effects are observed in subjects presenting Gilbert’s syndrome. This is defined as primary mild hyperbilirubinemia, i.e., the absence of hemolytic and/or liver diseases. It is an autosomal recessive hereditary condition found in about 7% of the European population and is asymptomatic. The molecular defect consists of mutations in the gene-encoding uridine diphosphate glycosyltransferase 1 (UGT1A1) [153,154]. Subjects carrying this genetic signature are shown to have improved endothelial function [155], fewer cardiovascular disease events [156], improved serum lipid profile [157], presumably enhanced lipid catabolism rate [158], lower inflammatory biomarkers [159] and lower risk of death [160].

#### 4.4.3. Bilirubin: Mechanistic Studies

Bilirubin is an antioxidant both in itself [161,162,163,164] and acting indirectly as it reduces the production of reactive oxygen species by the ubiquitous membrane enzyme NADPH oxidase (E.C. 1.6.3.1) [165,166]. Bilirubin acts as an electrophile binding to protein thiol groups, thus regulating the cellular redox state [167], and as a scavenger of reactive oxygen species, as demonstrated in in vivo mice [84]. It also has antibacterial activity against pathogenic bacteria of the gut microbiota [168]. Molecular studies indeed identified bilirubin as a ligand of several nuclear and cytoplasmic receptors, such as the aryl hydrocarbon receptor (AhR), the peroxisome proliferator-activated receptor-alpha (PPARα), the constitutive androstane receptor (CAR) and pregnane X receptor (PXR) and some others, as reviewed in Ref. [169]. These multiple functions result in fine regulation of metabolic genes related to lipid catabolism and glucose metabolism, especially in the liver [170].

The full spectrum of the bilirubin molecular targets remains to be explored.

## 5. Perspectives

At this stage of technological maturity, we can consider that the HUG-based assay for the analysis of bilirubin, bilirubin glucuronide and biliverdin can be regarded as an affordable emerging technology [171] that can not only enhance the analytical arsenal of laboratories active in life sciences but also add value to clinical diagnostics when an accurate quantification of each bile pigment is necessary and HPLC methods are the ultimate resource, as seen in Ref. [4].

Some limitations need more time and effort to be resolved. At present, the HUG biopolymer is not commercially available. This may limit its wide use and slow down its progress to TRL 7 and beyond. Though it can be easily produced by a standardized procedure at a low cost, its wide use is limited; thus, it must progress to TRL 7 and beyond. A corollary to this not-insurmountable limitation is that the HUG assay is so far intended to be operated only in laboratory settings in the pre-clinical or clinical research domains. At present, the HUG assay serves as a companion diagnostic device for precision medicine, given its accuracy and specificity in detecting three bile pigments. A larger number of assay demonstrations in addressing the significance of bile pigments as biomarkers of disease risk is needed to consolidate its position at TRL 6 and therefore move to industrial production (TRL 7), starting the procedures for regulatory approval (TRL 8).

Further cycles of technology innovation by advanced engineering approaches are needed to integrate HUG into microfluidic devices for use as a biomarker sensor in in vitro micro-physiological systems (e.g., organ-on-chip) or in portable, non-invasive analytical devices to measure bilirubin [172,173].

Improvements attainable in the short–medium term are possible due to the versatile properties of the HELP domain that enable the formation of composite materials [1,174,175], its shaping to 3D configuration by cross-linking [176] and its modulable thermoresponsive properties [48]. For example, it is possible to prototype HUG-coated multi-well plates or microfluidic systems to engineer composite biomaterials and develop multi-sensor devices by fusing the HELP scaffold with other on-demand functional domains [177].

At its present stage of technological readiness, the HUG assay is a reliable and invaluable resource for screening clinical biobanks of serum and other biofluids, as well as of other biological materials (e.g., experimental and livestock animals; samples of toxicological in vitro and in vivo tests), providing an immediate opportunity to step toward TRL 7 and beyond.

## Figures and Tables

**Table 1:** Optimized conditions for HELP expression in *E. coli* [27].

Condition	Specification
Medium and Supple	TB, terrific broth PGB, Phosphate buffer with glycerol
Recombinant System	NEBExpress^®^Iq Competent *E. coli* (High Efficiency)
Induction OD600	0.9–1
IPTG final concentration	0.1 mM
Growth after induction	4 h
Recombinant protein yield	180 mg/L

**Table 2:** Inverse transition temperatures of HELP at different solution conditions obtained by turbidimetric measurements.

C mg/mL	Solution	T_t_ °C
2.0	PBS, pH = 7.4	33
2.0	Tris, pH = 8.0 Tris, pH = 8.0, 0.15 M NaCl	30 36
2.0	NaPi, pH = 7.3 NaPi, pH = 7.3, 0.15 M NaCl	30 39

**Table 3:** Differential scanning calorimetry results of HELP solutions in different solvent conditions.

Solvent	C mg/mL	T_t_ °C	ΔH_tr_ kJ/mol	ΔS_tr_ J/molK
PBS pH = 7.4	4	34	216	23.9
10 mM Tris pH = 8 10 mM Tris pH = 8, 0.15 M NaCl	8 8	29 34	198 35	655 114
10 mM NaPi pH = 6.8 10 mM NaPi pH = 6.8, 0.15 M NaCl	8 8	29.5 39.5	6.67 0.141	21.8 0.45

**Table 4:** Synopsis of uses of UnaG in experimental biology and medicine.

Application	Purification Method	References
Bile pigment detection in biological fluid, cells and medium	-	[67,70,71,72,73,74,75]
	Ni^2+^ or glutathione-affinity chromatography	[76]
	Ni-NTA Fast Start kit or similar	[77,78,79]
Live-cell imaging	Anti-FLAG immuno-purification from cell lysate	[80]
	Ni^2+^-NTA agarose beads	[81]
	-	[77,82,83,84,85,86,87,88]
Lumirubin detection in urine	Ni^2+^ or glutathione-affinity chromatography	[3,89,90]
Live-cell imaging in bacteria	MBP-trap and His-trap	[91,92,93,94,95]
Bilirubin in tissues	Crude bacterial lysate (no further purification)	[96,97]
Probe for yeast display	-	[98]
BVR assay	Ni^2+^-NTA Fast Start kit	[79]
As gene reporter	-	[99,100,101,102,103,104,105,106,107,108]
Probe for anoxic/hypoxic conditions	-	[69,109,110,111,112,113,114,115,116,117,118,119]
Fluorescent probe with reversible switching in cells and tissues	GST-purification; ion exchange Chr; size-exclusion chr	[120]
Structural and functional studies	Ni^2+^ or glutathione-affinity chromatography	[121]
	MBP-trap and His-trap; gel filtration	[84]
	His-trap and desalted with a PD-10 column	[66]
	-	[122,123]

**Table 5:** Validation parameters of the HUG assay in the range 0.05–50 nM using standard bilirubin solutions containing 0.4 g/L BSA.

Validation Parameter		Value
Linearity	range	0–75 nM
	slope	785
	R^2^	0.9990
LOD	0.05–10 nM	0.36 nM
	0.5–50 nM	1.56 nM
LOQ	0.05–10 nM	1.10 nM
	0.5–50 nM	4.75 nM
Accuracy (relative error)	range	1–9%
	median	4.5%
Precision with standard solutions (coefficient of variation)	range	1.7–5.8%
	median	2.6%
Precision with human plasma (coefficient of variation)	range	1.9–12.6%
	mean	6.7%
